# Clinical and Radiological Markers of Extra-Motor Deficits in Amyotrophic Lateral Sclerosis

**DOI:** 10.3389/fneur.2018.01005

**Published:** 2018-11-22

**Authors:** Foteini Christidi, Efstratios Karavasilis, Michail Rentzos, Nikolaos Kelekis, Ioannis Evdokimidis, Peter Bede

**Affiliations:** ^1^First Department of Neurology, Aeginition Hospital, National and Kapodistrian University of Athens, Athens, Greece; ^2^Second Department of Radiology, University General Hospital Attikon, National and Kapodistrian University of Athens, Athens, Greece; ^3^Computational Neuroimaging Group, Academic Unit of Neurology, Trinity College Dublin, Dublin, Ireland

**Keywords:** amyotrophic lateral sclerosis, extra-motor involvement, cognition, behavior, neuropsychological deficits, neuroimaging

## Abstract

Amyotrophic lateral sclerosis (ALS) is now universally recognized as a complex multisystem disorder with considerable extra-motor involvement. The neuropsychological manifestations of frontotemporal, parietal, and basal ganglia involvement in ALS have important implications for compliance with assistive devices, survival, participation in clinical trials, caregiver burden, and the management of individual care needs. Recent advances in neuroimaging have been instrumental in characterizing the biological substrate of heterogeneous cognitive and behavioral deficits in ALS. In this review we discuss the clinical and radiological aspects of cognitive and behavioral impairment in ALS focusing on the recognition, assessment, and monitoring of these symptoms.

## Introduction

Amyotrophic lateral sclerosis (ALS) is the most common form of motor neuron disease (MND), a progressive neurodegenerative condition defined by concomitant lower and upper motor neuron degeneration (1). Motor symptoms include muscle weakness, fasciculations, cramps, as well as spasticity and brisk reflexes that accrue to considerable limb and bulbar disability over time, and eventually respiratory failure (1). The identification of TAR DNA-binding protein 43 (TDP-43) positive ubiquitinated cytoplasmic inclusions in almost all patients with ALS and more than half of patients with frontotemporal dementia (FTD) has placed ALS on the so-called “ALS-FTD continuum,” highlighting the considerable clinical, pathophysiological, and neuroimaging overlap between the two neurodegenerative conditions (2).

Although mentioned in early descriptions of ALS (3, 4), cognitive and behavioral deficits and frank dementia were previously considered atypical of ALS. It is not until the end of the twentieth century that clinical and research interest shifted to the extra-motor features of ALS and it has been gradually recognized as a genuine multisystem disease (5–8).

Neuropsychological deficits in ALS range from mild impairment to full-blown FTD. Up to 65% of ALS patients exhibit some cognitive or behavioral impairment (9–12) and 6–15% of sporadic ALS patients meet diagnostic criteria for FTD (10–13). While hexanucleotide repeat expansions in *C9ORF72* are often associated with ALS-FTD (14), extra-motor symptoms are not unique to this mutation and extra-motor neuroimaging findings can also be readily identified in a significant proportion of C9 negative patients (15, 16). The early recognition of extra-motor involvement in ALS is crucial due to its impact on functional decline (17), survival (18), compliance with assistive devices (19), decision-making, and engagement in end-of-life and legal decisions (20).

## Cognitive dysfunction

Much attention has been initially focused on executive dysfunction (21, 22) in ALS which has been gradually complemented by the characterization of language (23, 24), memory (25, 26), praxis (27), and theory of mind deficits (28) (Table 1). Population-based studies have confirmed distinct cognitive phenotypes without executive impairment (10, 11, 29).

**Table 1:** Most characteristic neuropsychological deficits in ALS categorised per cognitive domain.

**Main cognitive domains**	**Target processes/main deficits**	**Representative studies (First author, year, sample size ALS/Control)**
Executive functions	Verbal fluency	Ludolph, 1992 (21/12); Kew, 1993 (16/16); Abrahams, 1995 (12/6); Massman, 1996 (146/–); Abrahams, 1996 (12/6); Abrahams, 1997 (52/28); Frank, 1997 (74/56); Rakowicz, 1998 (18/24); Abrahams, 2000 (21/25); Lomen- Hanagasi, 2002 (20/13); Hoerth, 2003 (44/–); Abrahams, 2004 (28/18); Abrahams, 2005 (20/18); Pinkhardt, 2008 (20/20); Wicks, 2009 (41/35); Witgert, 2010 (225/–); Stukovnik, 2010 (22/21); Phukan, 2012 (160/110); Taylor, 2013 (51/35)
	Concept formation and mental flexibility	Abrahams, 1996 (12/6); Massman, 1996 (146/-); Abrahams, 1997 (52/28); Frank, 1997 (74/56); Evdokimidis, 2002 (51/28); Moretti, 2002 (14/15); Lomen-Hoerth, 2003 (44/-); Schreiber, 2005 (52/-); Libon, 2012 (41/25); Zalonis, 2012 (48/47); Taylor, 2013 (51/35)
	Mental set shifting	Hartikainen, 1993 (24/26); Hanagasi, 2002 (20/13); Kilani, 2004 (18/19); Witgert, 2010 (225/–)
	Response inhibition and attentional control	Abrahams, 1997 (52/28); Frank, 1997 (74/56); Hanagasi, 2002 (20/13); Moretti, 2002 (14/15); Lomen-Hoerth, 2003 (44/-); Sterling, 2010 (355/-); Christidi, 2012 (22/22); Phukan, 2012 (160/110); Zalonis, 2012 (48/47)
	Working memory	Abrahams, 1997 (52/28); Rakowicz, 1998 (18/24); Abrahams, 2000 (21/25); Hanagasi, 2002 (20/13); Abrahams, 2004 (28/18); Abrahams, 2005 (20/18); Lillo, 2012 (20/18)
	Reasoning and coordinating rules using ecologically valid measures	Meier, 2010 (18/18); Stukovnik, 2010 (22/21)
Memory	Episodic memory encoding	Hanagasi, 2002 (20/13); Mantovan, 2003 (20/20); Christidi, 2012 (22/22)
	Episodic memory retrieval	Hanagasi, 2002 (20/13); Ringholz, 2005 (279/129); Christidi, 2012 (22/22); Elamin, 2013 (186/120); Raaphorst, 2015 (26/21)
	Episodic memory consolidation/recognition	Machts, 2014 (40/40); Christidi, 2012 (22/22)
	Visual delayed recall	Ringholz, 2005 (279/129)
	Semantic memory	Hervieu-Begue, 2016 (15/-)
Language	Verb naming and action verb processing	Bak, 2001 (6/20); Grossman, 2008 (34/25); York, 2014 (36/13); Papeo, 2015 (21/14)
	Grammatical errors	Ash, 2015 (26/19); Tsermentseli, 2015 (26/26)
	Phonemic and semantic paraphasias	Roberts-South, 2012 (16/12); Tsermentseli, 2015 (26/26)
	Establishing and adhering to the main topic of conversations	Ash, 2015 (26/19); Bambini, 2016 (33/33)
	Narrative speech pauses	Yunusova, 2016 (85/33)
	Syntactic processing/comprehension	Yoshizawa, 2014 (25/–); Tsermentseli, 2015 (26/26); Kamminga, 2016 (35/23)
Praxis	Constructive apraxia	Abrahams, 1997 (52/28)
	Orofacial apraxia	Lobo, 2013 (1/–)
	Speech apraxia	Duffy, 2007 (7/–)
	Respiratory apraxia	Pinto, 2007 (1/–)
Social cognition	Theory of mind	Meier, 2010 (18/18); Girardi, 2011 (19/20); Burke, 2016 (59/59)
	Emotional processing and ability to recognize emotional facial expressions	Palmieri, 2010 (9/10); Girardi, 2011 (19/20); Crespi, 2014 (22/55); Savage, 2014 (29/30); Andrews, 2017 (33/22)
	Ability to describe intentions and feelings of others	Gibbons, 2007 (16/16); Staios, 2013 (35/30); Cerami, 2014 (20/56)
	Empathy	Girardi, 2011 (19/20); Cerami, 2014 (20/56)
	Social inferences	Staios, 2013 (35/30); Savage, 2014 (29/30)
Behavior	Apathy	Grossman, 2007 (45/–); Chio, 2010 (70/–); Witgert, 2010 (225/–); Girardi, 2011 (19/20); Radakovic, 2016 (83/83)
	Disinhibition	Grossman, 2007 (45/–); Terada, 2011 (24/–)
	Pathological crying and laughing	McCullagh, 1999 (18/10); Palmieri, 2009 (32/39); Olney, 2011 (35/–); Brooks, 2013 (9/–); Floeter, 2014 (22/28); Christidi, 2018 (56/25)

*ALS, amyotrophic lateral sclerosis*.

### Executive dysfunction

Executive dysfunction is the most commonly cited facet of cognitive impairment in ALS. Executive function however is an umbrella term encompassing several relatively distinct higher-order processes, such as planning, organization, goal-directed activity, working memory, initiation, behavioral regulation, and inhibitory control, as well as situation-appropriate decision-making on the basis of projected positive and negative outcomes in novel, complex or ambiguous situations (30). In addition, tests of verbal (i.e., phonemic and semantic/category) and figural/design fluency are also often conceptualized as proxies of executive performance (31).

Verbal fluency impairment has been consistently reported in ALS (11, 22, 24, 27, 32–46). Coexisting phonemic and semantic fluency dysfunction or phonemic fluency deficits alone are often linked to executive dysfunction, while isolated semantic fluency deficits are associated with impaired semantic memory processing. Semantic (24, 34, 40, 44, 46, 47) and figural (34, 46) fluency are not typically impaired in ALS. A verbal fluency index has been proposed and is now widely utilized to account for patients' motor disability (32, 48). Other executive processes are also affected in ALS, such as concept formation and mental flexibility (24, 27, 33, 35, 36, 41, 49–53) which is typically examined by the Wisconsin Card Sorting Test or the Dellis-Kaplan Executive Function System Card Sorting Test (31). However, not all neuropsychology studies corroborate these findings (34, 38, 39, 45, 46, 54–56). Several studies have specifically evaluated mental set shifting ability in ALS using the Trail Making Test; most of them identifying considerable dysfunction (37, 42, 47, 55, 57), while others have not captured such deficits (58, 59). Response inhibition and attentional control are typically examined by the Stroop test, and are often impaired in ALS (11, 27, 35–37, 51, 53, 57, 60), but unaffected cohorts have also been reported (39, 40, 44, 49). ALS patients also often exhibit difficulties in maintaining, manipulating and retrieving information relying on working memory (27, 32, 34, 37, 43, 46, 61), but preserved working memory has also been observed (39, 44, 51, 54, 55, 58, 62). Subtle deficits in reasoning and coordinating rules have been found using ecologically valid measures of executive functions (44, 63).

### Memory deficits

Following inconsistent initial reports, memory dysfunction in ALS has received increasing attention recently (7, 64). While autobiographic memory seems to be preserved in ALS (65), semantic memory is often affected (66). Episodic memory is the most commonly evaluated memory domain in ALS, typically tested by list-learning tests, associate-learning tests, prose memory, as well as visual memory tests (7). Several studies have reported mild to moderate episodic memory impairments which are often interpreted as the corollary of underlying executive deficits (27, 35, 37, 39, 41, 67–69). Memory impairment in ALS is rarely identified in isolation (11), but using data-driven taxonomy approaches a subgroup of patients may show non-executive memory dysfunction (29). Several studies have found impaired encoding (37, 60, 68), retrieval (12, 17, 37, 60, 70) consolidation and recognition (26, 60), although recognition deficits in ALS are not universally recognized (11, 37, 41). Visual memory dysfunction has also been noted in ALS (12), although visual recall is typically less affected than delayed verbal recall (7). Neuroimaging studies have contributed to the characterization of ALS-associated memory impairment highlighting mesial temporal lobe involvement irrespective of frontal lobe pathology (64).

### Language deficits

Language deficits in ALS have traditionally attracted less attention compared to other cognitive domains and have been mostly appraised in association with ALS-FTD (7, 23, 71, 72). However, language dysfunction is increasingly recognized as a core feature of ALS and has been consistently detected in patients without executive dysfunction (24, 29, 73). Patients with ALS show impaired syntactic processing (74), deficits in verb naming and action verb processing (75, 76). Selective impairment in action knowledge (77) has been directly associated with motor cortex degeneration (78) suggesting a link between action execution and action conceptualization (79). Grammatical errors such as incomplete utterances (73, 74) and omission of determiners (73) have been reported in ALS and seem to be dissociable from the patients' motor and executive deficits (73). Phonemic and semantic paraphasias have also been reported (74, 80). Patients with ALS may find narrative discourse particularly challenging due to difficulties to establish (81) and adhere to the main topic of conversation (73, 81). Frequent pauses are another key characteristic of narrative speech in ALS in both demented and non-demented ALS cohorts (82). Syntactic comprehension deficits have also been detected in up to 72% of patients with ALS (83, 84).

### Visuo-perceptive and visuo-constructive deficits

Visuo-perceptive and visuo-constructive functions are seldom specifically examined in ALS. Existing studies tend to focus on visuospatial memory measures and often fail to reach definite conclusions (37, 41, 46, 47, 85). Based on large meta-analyses, these domains are not significantly affected in ALS (7). The relative absence of visuo-perceptual deficits is further supported by the lack of reports on Balint's syndrome in ALS and is consistent with limited occipital involvement on neuroimaging (86) and pathology (87). While praxis deficits are also rarely reported in ALS (27), orofacial (88), speech (89), and respiratory (90) apraxia have been sporadically reported.

### Social cognition deficits

Social cognition refers to a diverse set of cognitive skills that allow humans to understand themselves, interact with and understand others and are crucial to adopt situation-appropriate, goal-directed behaviors in everyday social interactions (91). Despite considerable variations, deficits in theory of mind, empathy, social perception, social behavior are now recognized as key elements of the ALS-associated cognitive profile (7, 28, 92). It is however still unclear if these deficits are linked to executive dysfunction (29, 93–98) or may be related to non-executive domains, such as episodic memory function and visuospatial abilities (99). Patients with ALS may also exhibit impaired emotional processing and ability to interpret emotional facial expressions, especially with comorbid FTD (96, 100–102). Impairments in complex facial affect recognition, affective prosody recognition and cross-modal integration have also been found in non-demented ALS cohorts (103). Multiple subcomponents of theory of mind seem to be affected in ALS, including the ability to describe the intentions and feelings of others (95, 98, 104), to recognize and provide explanations for social “faux pas” (63) and evaluate object preferences based on the interpretation of eye gaze direction (96, 105). Loss of empathy (96), impaired emotional empathy attribution (95), and erroneous social inferences (98, 100) have also been reported in non-demented ALS cohorts.

### Behavioral deficits

The clinical link between ALS and FTD is exemplified by overlapping behavioral changes which are similar to those observed in behavioral variant of FTD (106). These deficits are typically identified through a structured clinical interview with the caregivers or through validated questionnaires. Perseveration, apathy and disinhibition are the most commonly reported behavioral alterations, followed by loss of disease insight, indifference, loss of interest, aggression, irritability, and lability (107).

Apathy is the most commonly reported behavioral symptom in non-demented ALS (42, 45, 96, 108, 109), which used to be assessed by generic behavioral instruments, such as the Frontal Systems Behavior Scale (110) and the Frontal Behavioral Inventory (111), until the development of ALS-specific scales, such as the Dimensional Apathy Scale (112) which appraises initiation, executive and emotional apathy. Initiation apathy is thought to be particularly prevalent in ALS (113). ALS patients with apathy may require prompts to initiate or follow through with a task, including self-care, feeding, and taking medications. They may appear poorly motivated, aloof or uninterested. Apathy may impact of rehabilitation, hamper gait initiation, and curb communication efforts especially in the presence of bulbar impairment. It can be mistaken for low mood, depression and withdrawal by inexperienced observers. Disinhibition is more readily identified and reported by caregivers, and can precede (108) or follow (114) motor disability. Disinhibited behavior can manifest in rude, offensive, flirtatious comments, puns, “Witzelsucht” often violating social norms, personal space and may result in careless or impulsive decisions. Purchasing expensive items on a whim, hoarding, compulsive behavior, overeating, and developing a preference for sweets have also been reported (115).

Hallucinations have been reported by several groups (116–119) and are sometimes associated with the C9orf72 genotype. Symptomatic treatment includes the judicious use of small dose atypical antipsychotics, if necessary.

Patients with pseudobulbar affect or pathological crying and laughing exhibit sudden situation-inappropriate emotional responses (120–122) which may have a negative impact on their quality of life (123) and lead to social isolation or social stigma. It is most commonly associated with UMN-type bulbar dysfunction (124), but frontal abnormalities, executive dysfunction, basal ganglia pathology and impaired cerebellar gating mechanisms have also been linked this symptom (27, 122, 125–128).

## Insights from neuroimaging

Neuroimaging techniques provide optimal non-invasive tools to characterize extra-motor pathology in ALS underpinning cognitive and behavioral deficits and also permit exploratory correlations with clinical measures (129, 130).

### Structural imaging

Voxel based morphometry (VBM) and surface-based morphometry (SBM) are reproducible, validated and widely-used pipelines that use high resolution 3D T1-weighted MR images to identify focal GM alterations. Beyond the consensus on motor cortex atrophy (131), many studies also detect multifocal frontotemporal and parietal GM changes (132). GM abnormalities have also been identified in subcortical structures (133), such as the hippocampus (134–136), amygdala (137, 138), thalamus (134, 135, 139, 140), and insula (141, 142). Reduced GM density in occipital (139, 143–145) and cerebellar (139, 146) regions is less commonly reported. GM alterations in extra-motor areas have been linked to structure-specific cognitive and behavioral deficits in ALS (147, 148). Recent studies have highlighted extra-motor cortical changes in ALS patients without overt cognitive impairment (134, 135, 146, 149, 150). The anatomical patterns of extra-motor gray matter involvement in ALS further support the notion of the ALS-FTD continuum (72).

White matter integrity in ALS is most commonly evaluated by diffusion tensor imaging (DTI). Reduced fractional anisotropy and increased axial and radial diffusivity in the corticospinal tracts and corpus callosum are hallmark features of ALS (151, 152). Extra-motor white matter pathology has been consistently detected in frontal (139, 153–160), temporal (53, 154, 161), cingular (162), parahippocampal (25, 157, 160), insular (160), thalamic (141, 159, 163), and cerebellar regions (86, 146, 164). Similarly to gray matter analyses, extra-motor white matter involvement has also been identified in ALS patients without overt cognitive impairment (146).

### Metabolic imaging

MR spectroscopy in ALS has consistently revealed decreased N-acetyl aspartate (NAA)/choline and NAA/creatine ratios in motor regions (165–167), but whole brain spectroscopy also detected extra-motor NAA reductions in frontal, parietal, thalamic and occipital areas (168, 169).

Most positron emission tomography (PET) studies in ALS use 18F-FDG PET, but TSPO, GABA_A_ (11C-flumazenil) and 5-HT1A receptor (11C-WAY100635) radioligands have also been utilized (170). Hypometabolism in motor regions is a characteristic FDG-PET finding in ALS (171–174), but extra-motor changes in dorsolateral prefrontal, orbitofrontal, anterior frontal, anterior temporal, fusiform, and occipital regions have also been reported (171–174). Frontotemporal hypometabolism has been linked to cognitive performance (22, 39, 172), is thought to precede atrophy (175) and has been linked to shorter survival (176). There is also evidence of hypermetabolism in the hippocampus, amygdala midbrain, pons and cerebellum (173, 174, 177). PET imaging has identified microglial activation in frontotemporal, thalamic, midbrain, and pontine regions suggestive of extra-motor inflammation (178–181). Widespread reduction of 11C-Flumazenil binding to GABA_A_ in sporadic ALS has been interpreted as inhibitory dysfunction (182) and is regarded as a one of cornerstones of ALS pathogenesis (183). Reduced serotonin receptor binding has also been reported in ALS using the 11C-WAY100635 radio-ligand (184).

### Functional imaging

Resting state fMRI enables the assessment of functional connectivity between different brain regions by evaluating synchronized neuronal activity at rest. Reduced (185–189) and increased (183, 190) functional connectivity have both been reported in sensorimotor networks of ALS patients which may be explained by the different sub-regions evaluated (191–193) and also by the inclusion of patients in different disease-stages. Similarly, both reduced and increased functional connectivity alterations have been reported in extra-motor areas which mediate cognitive and behavioral functions (187, 188, 193, 194). The functional connectivity of the default mode network (DMN) has been reported to be both decreased (187, 189, 193) and increased (193, 195). Increased functional connectivity has been detected in the DMN using graph theory-based analyses (196). Increased (193) and decreased (186, 189, 193) fronto-parietal network integrity has been both reported. Reduced “executive control network” (middle frontal cortex) and “salience network” (medial prefrontal cortex, insula) connectivity has been described in ALS cohorts without dementia (189). Increased connectivity in ALS has either been interpreted as evidence of attempted compensation for structural degeneration (197, 198) or proof of inhibitory dysfunction (183, 190, 199).

Task-based fMRI studies in ALS have consistently revealed the recruitment of pre- and supplementary motor regions when executing motor tasks. Additional activation has also been observed in areas associated with motor learning areas, such as the basal ganglia and cerebellum (200, 201). Despite difference in study protocols, an activation shift to premotor (202, 203), temporal and parietal regions (203–205) has been often noted. Cognitive paradigms have been particularly helpful in capturing frontotemporal network alterations. Impaired verbal fluency was linked to reduced frontotemporal, parietal, and cingulate activation in non-demented ALS patients (46). Impaired frontal inhibitory control was confirmed by a number of fMRI paradigms, such as Stroop, negative priming, antisaccade tasks, go/no-go tasks etc. Increased activation during the Stroop paradigm and decreased activation in negative priming conditions has been reported mostly in left hemispheric regions (206). Increased activation in supplementary and frontal eye fields and reduced activation in dorsolateral prefrontal cortex have been noted in antisaccade tasks (207). Furthermore, in go/no-go paradigms, ALS patients show increased inhibition-related activation in frontal and basal ganglia regions and increased execution-related activity in contralateral sensorimotor regions (208). Few studies have specifically examined the functional correlates of social cognition to date. Patients with ALS tend to show increased activation compared to healthy participants in the right supramarginal, anterior cingulate and bilateral dorsolateral prefrontal cortex in response to socio-emotional stimuli (56, 209). The combined use of motor and memory tasks on fMRI enables the longitudinal characterization of divergent motor and extra-motor functional changes. Increased motor activation was found in ALS compared to controls at baseline, which has decreased on the follow-up assessment, suggestive of failing compensation. Contrary to the functional motor changes, hippocampal activation increased on follow-up when novel stimuli was presented (210).

## Relevance to clinical care

The detection (48), expert evaluation (11), categorization (211), and follow-up (17) of extra-motor deficits in ALS is crucially important for individualized patient care. While screening tests (Table 2) are useful for the detection of gross deficits, expert review by neuropsychologists is indicated for accurate patient classification. Adherence to treatment, compliance with assistive devices, participation in clinical trials, making informed financial and end-of-life decisions, choices in participating in non-licensed treatments are just some of the aspects of a patient journey which may be significantly affected by cognitive or behavioral deficits (19, 212). Cognitive impairment in ALS is widely regarded as a negative prognostic indicator and linked to reduced survival (17, 18, 213). Neuropsychological deficits in ALS are thought to be associated with increased caregiver burden (214, 215) and reduced quality of life (216). The recognition of the far-reaching effects of neuropsychological deficits on nearly all aspects of ALS care, caregiver support, resource allocation, and prognosis, led to the inclusion of specialist neuropsychologists as core members of ALS multidisciplinary teams worldwide (217, 218). The careful evaluation of motor deficits which are not directly linked to the corticospinal axis and are not reflected in the ALSFRS-R score, such as extra-pyramidal deficits are also crucial (219). Extra-pyramidal deficits may contribute to falls and gait impairment and are increasingly investigated in neuroimaging studies (220, 221). These symptoms may present early in the course of the disease, and contribute the clinical heterogeneity of the condition (220, 222). Postural instability and rigidity may be associated with other extra-motor deficits, and potentially linked to poor survival (205, 223). There is some controversy about the chronology of motor and extra-motor involvement in ALS. Extra-motor manifestations, such as dementia (224, 225), psychiatric features (226), and extra-pyramidal symptoms (227) have been reported to precede motor symptoms in some cases, and there is also compelling evidence of early extra-motor pathology in cognitively normal ALS patients (134, 135, 146).

**Table 2:** ALS-specific instruments to screen for cognitive and behavioral changes at baseline and during the course of the disease.

**Screening instrument**	**Duration of administration**	**Cognitive and behavioral domains examined**	**Parallel forms for longitudinal assessment**	**Validation in non-English speaking populations**
Edinburgh Cognitive and Behavioral ALS Screen (ECAS)	15–20 min	Executive functions, Social cognition, Language, Visuoconstruction, Memory Behavioral changes (including psychotic symptoms)	Yes	American-English; Belgium; Chinese; Croatian; Czech; Dutch; French; German; Swiss-German; Greek; Hebrew; Italian; Japanese; Norwegian; Polish; Portuguese; Russian; Slovak; Slovenian; Spanish; Swedish; Welsh
ALS Cognitive and Behavioral Screen (ALS-CBS)	<10 min	Executive functions including attention, concentration, mental tracking and monitoring, verbal fluency Behavioral changes	Yes	Brazilian; Spanish; Greek
ALS Brief Cognitive Assessment (ALS-BCA)	5 min	Executive functions (working memory, set-shifting), Frontally-mediated language function, Delayed verbal recall, Behavioral changes	N/A	N/A
Beaumont Behavioral Inventory (BBI)	5–10 min	Frontal Behavioral symptoms; Executive functions; Language; Psychotic symptoms	N/A	N/A
Motor Neuron Disease Behavioral Instrument (MiND-B)	<10 min	Behavioral symptoms	N/A	N/A
ALS Frontotemporal Dementia Questionnaire (ALS-FTD-Q)	5–10 min	Behavioral symptoms (it also includes 3 items for memory, concentration and orientation in time)	N/A	N/A

## Research opportunities and future directions

Even though the high incidence of cognitive impairment and its impact on individualized patient care are now universally recognized, the neuropsychological aspects of ALS are seldom considered for patient stratification in clinical trials (228). Several ALS-specific cognitive screening tests have now been validated, but generic tests, such as MOCA and MMSE are still in use in some clinics. While neuropsychological scores are often adjusted for motor-disability and depression, medication-effects, fatigue, and hypoxia are seldom considered when interpreting cognitive performance on various instruments. Despite sporadic reports, the full spectrum of psychiatric manifestations and the precise incidence of psychosis remain to be established in ALS (119, 229, 230). Certain cognitive domains, such as memory and praxis have not been exhaustively characterized in ALS to date. Relatively little is known of the neuropsychological profile of ALS-causing mutation carriers before they develop motor symptoms (231–233). The gaps in our current understanding of extra-motor pathology in ALS shape future study designs. Novel technologies such as online assessments, internet-based data collection, mobile phone apps, and wearable devices are emerging research resources. Irrespective of specific neuropsychological instruments, the early detection, and careful of monitoring of cognitive deficits in ALS is pivotal for optimized patient and caregiver support and tailoring precision management strategies to individual patient needs.

## Author contributions

The paper was drafted by FC, EK, and PB and has been reviewed for intellectual content by MR, NK, and IE.

### Conflict of interest statement

The authors declare that the research was conducted in the absence of any commercial or financial relationships that could be construed as a potential conflict of interest.
